# Bequests to Children

**Published:** 1882-07

**Authors:** 


### BEQUESTS TO CHILDREN.

SOME one has said, give your children a fortune without education, and at least one half the number will go to ruin. This is but part of a great truth. Give your children a fortune *and* an education, without instilling those religious principles which come from the warm heart and loving lips of a pious mother, and those children will, in the large majority of cases, grow up to an aimless life, to early ruin here, and perdition hereafter. It is too much the fashion now-a-days to deify "Education," to make it the panacea for all human ills; but without the accompaniment of sterling religious principle, it is but a ship in a storm, without a rudder on a rock-bound coast, an engine of death in giant and reckless hands.

SUDDEN DEATH.—The chances of escaping sudden death are nearly two to one in favor of women. Death always begins at the head, the heart or the the lungs; therefore,

1. Keep the head cool by taking the world easy.
2. Keep the lungs breathing deeply and fully about seventeen times a minute, by cultivating alacrity in all the bodily movements.
3. Keep the heart beating about sixty-eight times a minute, that is, let the pulse beat four times while the lungs breathe once, by eating temperately, sleeping fully and soundly, exercising moderately, and avoiding all temporary excitants, mental or liquid.

